# Highly variable biological effects of statins on cancer, non-cancer, and stem cells in vitro

**DOI:** 10.1038/s41598-024-62615-w

**Published:** 2024-05-23

**Authors:** Helena Gbelcová, Silvie Rimpelová, Adriana Jariabková, Patrik Macášek, Petra Priščáková, Tomáš Ruml, Jana Šáchová, Jan Kubovčiak, Michal Kolář, Libor Vítek

**Affiliations:** 1 Institute of Medical Biology, Genetics and Clinical Genetics, Faculty of Medicine, Comenius University, Bratislava, 813 72 Slovak Republic; 2 Department of Biochemistry and Microbiology, University of Chemistry and Technology, Prague, 166 28 Czech Republic; 3 Laboratory of Genomics and Bioinformatics, Institute of Molecular Genetics, Czech Academy of Sciences, Prague, 142 20 Czech Republic; 4 Department of Informatics and Chemistry, University of Chemistry and Technology, Prague, 166 28 Czech Republic; 5 Institute of Medical Biochemistry and Laboratory Diagnostics, and 4Th Department of Internal Medicine, 1St Faculty of Medicine, Charles University and General University Hospital in Prague, Prague, 121 08 Czech Republic

**Keywords:** Pancreatic cancer cells, Stem cells, Microarray analysis, Cell spheroids, 3-Hydroxy-3-methylglutaryl coenzyme A inhibitors, Statins, Genetics, Molecular biology

## Abstract

Statins, the drugs used for the treatment of hypercholesterolemia, have come into the spotlight not only as chemoadjuvants, but also as potential stem cell modulators in the context of regenerative therapy. In our study, we compared the in vitro effects of all clinically used statins on the viability of human pancreatic cancer (MiaPaCa-2) cells, non-cancerous human embryonic kidney (HEK 293) cells and adipose-derived mesenchymal stem cells (ADMSC). Additionally, the effect of statins on viability of MiaPaCa-2 and ADMSC cells spheroids was tested. Furthermore, we performed a microarray analysis on ADMSCs treated with individual statins (12 μM) and compared the importance of the effects of statins on gene expression between stem cells and pancreatic cancer cells. Concentrations of statins that significantly affected cancer cells viability (< 40 μM) did not affect stem cells viability after 24 h. Moreover, statins that didn´t affect viability of cancer cells grown in a monolayer, induce the disintegration of cancer cell spheroids. The effect of statins on gene expression was significantly less pronounced in stem cells compared to pancreatic cancer cells. In conclusion, the low efficacy of statins on non-tumor and stem cells at concentrations sufficient for cancer cells growth inhibition, support their applicability in chemoadjuvant tumor therapy.

## Introduction

Despite significant advances in the medical science, there is still a large number of pathological conditions, such as degenerative and cancer diseases, that cannot be satisfactorily treated with standard therapies. Therefore, alternative strategies that would lead to the restoration of damaged or degenerated tissue or that would contribute as an adjuvant therapy to conventional treatment have been searched. In this sense, the application of stem cells today appears to be the most progressive therapeutic method^1,2^. An enormous number of studies have been published on the possibility of inducing stem cell differentiation to desired tissue types^3^. The most limiting factor of stem cell therapy represents the risk of disorganized cell growth, proliferation, and division that possibly lead to tumor formation^4,5^. One of the potential alternatives to eliminate such risk is the application of statins^6^.

Statins are the dominant group of compounds used for the treatment of hypercholesterolemia and cardiovascular diseases^7^ due to their ability to inhibit de novo cholesterol synthesis. In total, eight statins have been introduced for clinical purposes: lovastatin, pravastatin, simvastatin, fluvastatin, atorvastatin, rosuvastatin, pitavastatin, and cerivastatin^8^. Although individual statins differ from each other in their chemical structure, physico-chemical properties, source or preparation, metabolism, etc., the common characteristic of all of them is the competitive inhibition of the rate-limiting step of the mevalonate pathway catalyzed by 3-hydroxy-3-methylglutaryl coenzyme A (HMG-CoA) reductase^9^. Due to the depletion of intermediates of the mevalonate pathway, statins have, in addition to hypolipidemic effects, several other pleiotropic biological effects that play an important role in preventing the progression of many clinical conditions, including cancer diseases^10^.

To apply individual statins, whether, in chemoadjuvant therapy of malignant tumors or regenerative medicine, it is necessary to know in detail the mechanism of their effect on proliferation and survival of not only cancer but also non-cancerous and stem cells. Therefore, we compared in this study the effect of individual statins on the survival of stem, cancer and non-cancerous cells grown in two-dimensional (2D) and stem and cancer cells grown in three-dimensional (3D) conditions (monolayer and spheroids, respectively), and using the whole genome / whole transcriptome microarray analyses we studied biological events that were most affected by individual statins.

## Results

### Comparison of in vitro effects of statins on viability and growth of stem, non-cancerous and cancer cells cultured in a monolayer

**Figure 1:**
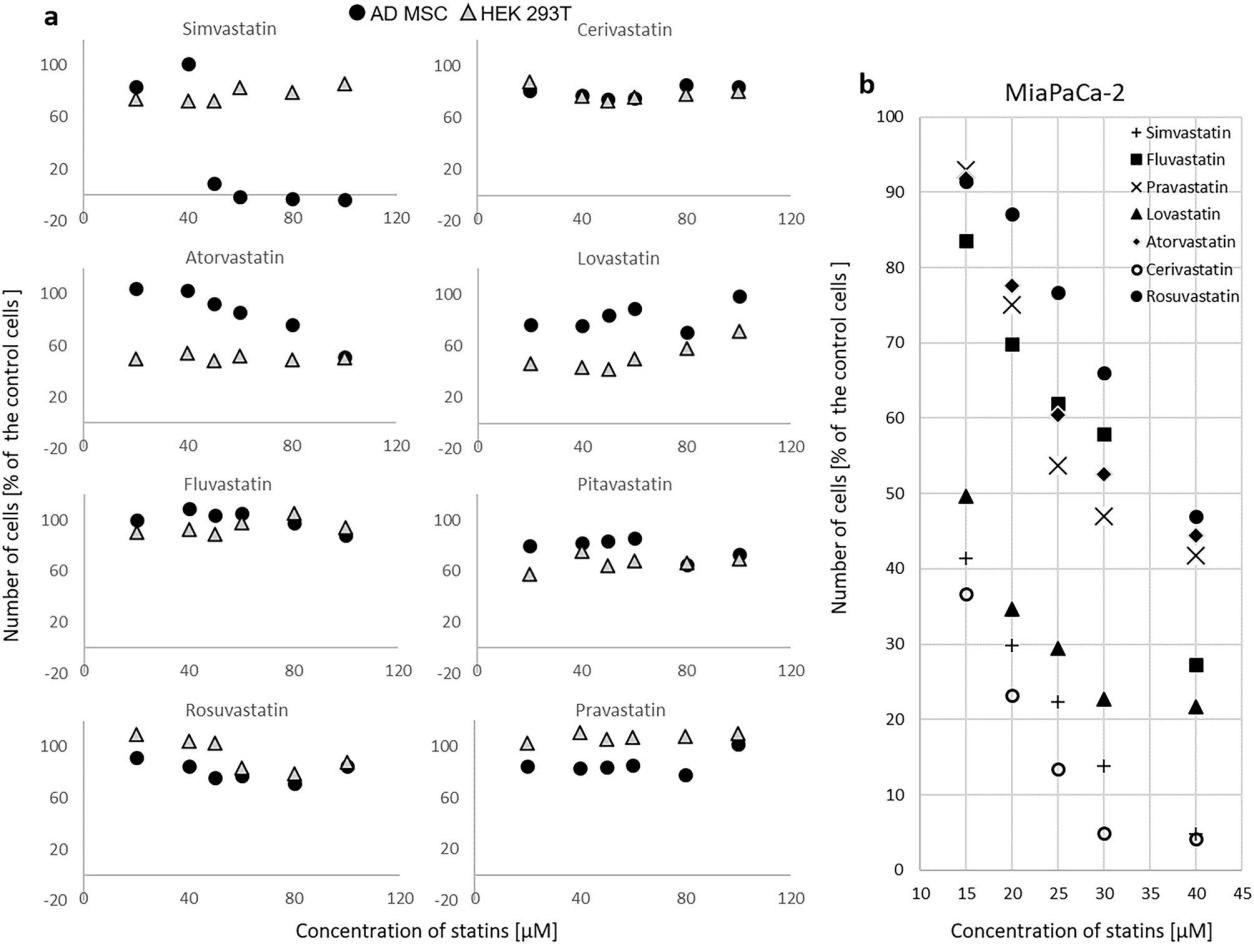
The effect of statins on viability and growth of (**a**) stem ADMSC and non-cancerous HEK 293 cells and (**b**) cancer MiaPaCa-2 cells. (**a**) ADMSC—human adipose-derived mesenchymal stem cells, HEK 293—human embryonic kidney cells, exposure to statins—24 h, concentrations 0—100 µM, control—methanol, (**b**) previously published data^11^, MiaPaCa-2—pancreatic cancer cells, exposure to statins—24 h, concentrations 0—40 µM, control—methanol.

Initially, non-cancerous cells HEK 293 and stem cells ADMSC were exposed to individual statins within a concentration range of 0–40 µM. This range was chosen based on the fact that IC_50_ values for all the statins as determined for pancreatic cancer cells MiaPaCa-2 were found to be less than 40 µM after 24 h of treatment^11,12^. As we did not detect any significant effect of statins on the viability and growth of ADMSC, the statin concentration range was extended to 100 µM concentration. Interestingly, even after 24 h of exposure to a statin concentration of 100 µM in cell growth medium, the number of stem cells did not decrease dramatically, except for simvastatin (Fig. 1). Simvastatin reduced the viability of ADMSC by more than 80% already at a concentration of 50 µM (p < 0.005). Compared to ADMSC, HEK 293 cells were only slightly more sensitive to the antiproliferative effect of certain statins (Fig. 1); with lovastatin, pitavastatin, and atorvastatin, the most pronounced antiproliferative effect was obtained, even at the lowest concentrations used (Fig. 1).

Interestingly, extending the incubation time of HEK 293 and ADMSC cells with statins to 48 and 72 h resulted in a growth inhibitory effect of statins (except for pravastatin and partially rosuvastatin), however, this effect was induced already by the lowest tested concentration of statins (20 µM), and was not significantly pronounced by increased concentration (data not shown). In contrast, in some cases (for example, cerivastatin, ADMSC, 48 h of incubation), the intensity of the antiproliferative effect of statin decreased with increasing concentration (data not shown).

**Figure 2:**
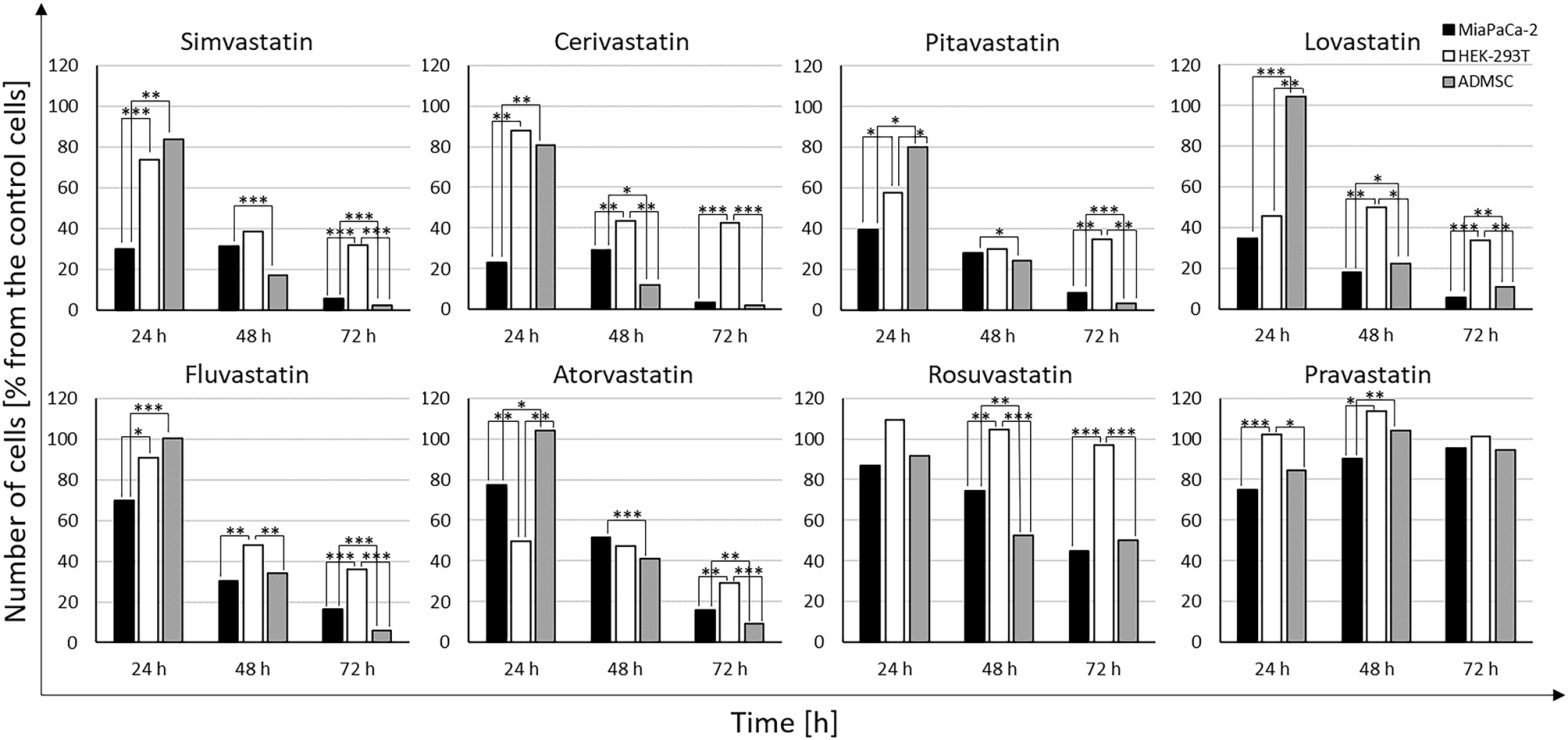
Comparison of the effect of statins on the growth and viability of pancreatic cancer MiaPaCa-2 cells, non-cancerous HEK 293 cells, and ADMSC stem cells. Concentration of statins—20 µM, Time—exposure to statins—24, 48, and 72 h, control—methanol.

The comparison of the effect of statins (20 µM) on the growth and viability of pancreatic cancer MiaPaCa-2 cells, non-cancerous HEK 293 cells, and ADMSC stem cells is shown in Fig. 2. Non-cancerous HEK 293 cells are more resistant to statins compared to pancreatic cancer MiaPaCa-2 cells even after 72 h, while a delayed effect was observed in ADMSC stem cells, similar to that observed in pancreatic cancer MiaPaCa-2 cells (Fig. 2).

### In vitro effect of statins on the size and compactness of spheroids of cancerous and stem cells

**Figure 3:**
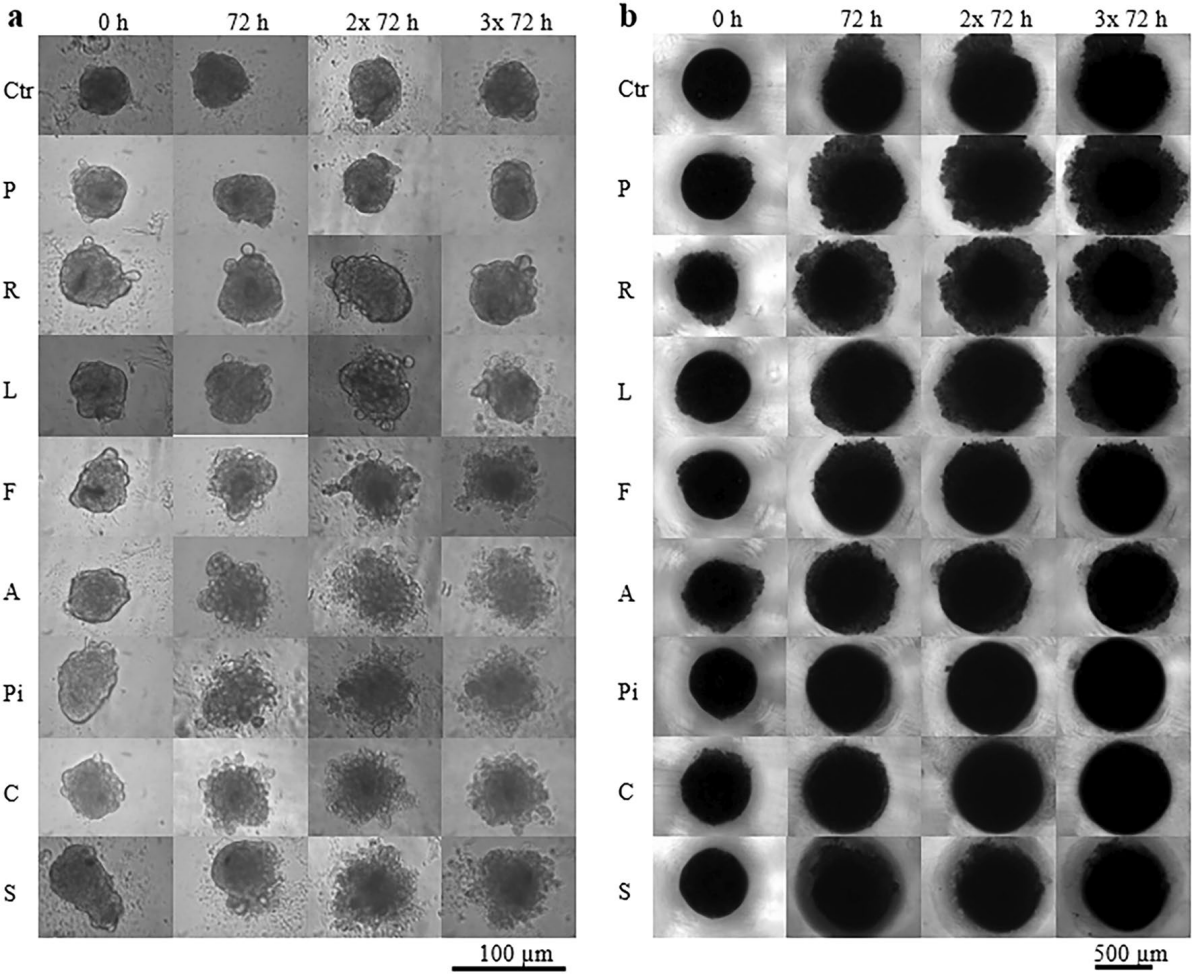
Effect of statins on size and compactness of spheroids. (**a**) ADMSC stem cells, (**b**) pancreatic cancer MiaPaCa-2 cells, concentration of statins—20 µM, Ctr—methanol treated spheroids, P—pravastatin, R—rosuvastatin, L—lovastatin, F—fluvastatin, A—atorvastatin, Pi—pitavastatin, C—cerivastatin, S—simvastatin. Statins were added once, after spheroid formation, 10 weeks (**a**) or 3.5 weeks (**b**) after inoculation. Experiment was carried out in biological dodecaplicates.

The control ADMSC spheroids formed from a small inoculum of stem cells did not significantly change their size during the nine-day observation period after the long-term cultivation (statins were added 10 and 3.5 weeks after the inoculation of ADMSC and MiaPaCa-2 cells, respectively) (Fig. 3a). However, the control spheroids of pancreatic cancer cells became observably larger during the nine-days observation period (Fig. 3b).

The less effective statins (pravastatin and rosuvastatin) had no visible/detectable effect on size and morphology of the ADMSC spheroids. Spheroids affected by other statins began to decay after the first 72 h of statin treatment (Fig. 3a).

Statins, which were less effective in the 2D arrangement did not prevent the further growth of pancreatic cancer cell spheroids; however, they reduced the compactness of spheroids compared to the control spheroids (Fig. 3b, pravastatin and rosuvastatin). Other statins did not induce changes in the compactness and size of the pancreatic cancer cell spheroids when compared to the control spheroids significantly enough to be evaluated by light microscopy (Fig. 3b). Interestingly, statins that affected stem cell spheroids did not affect cancer cell spheroids and vice versa.

### In vitro effect of statins on formation of spheroids of cancer and stem cells

**Figure 4:**
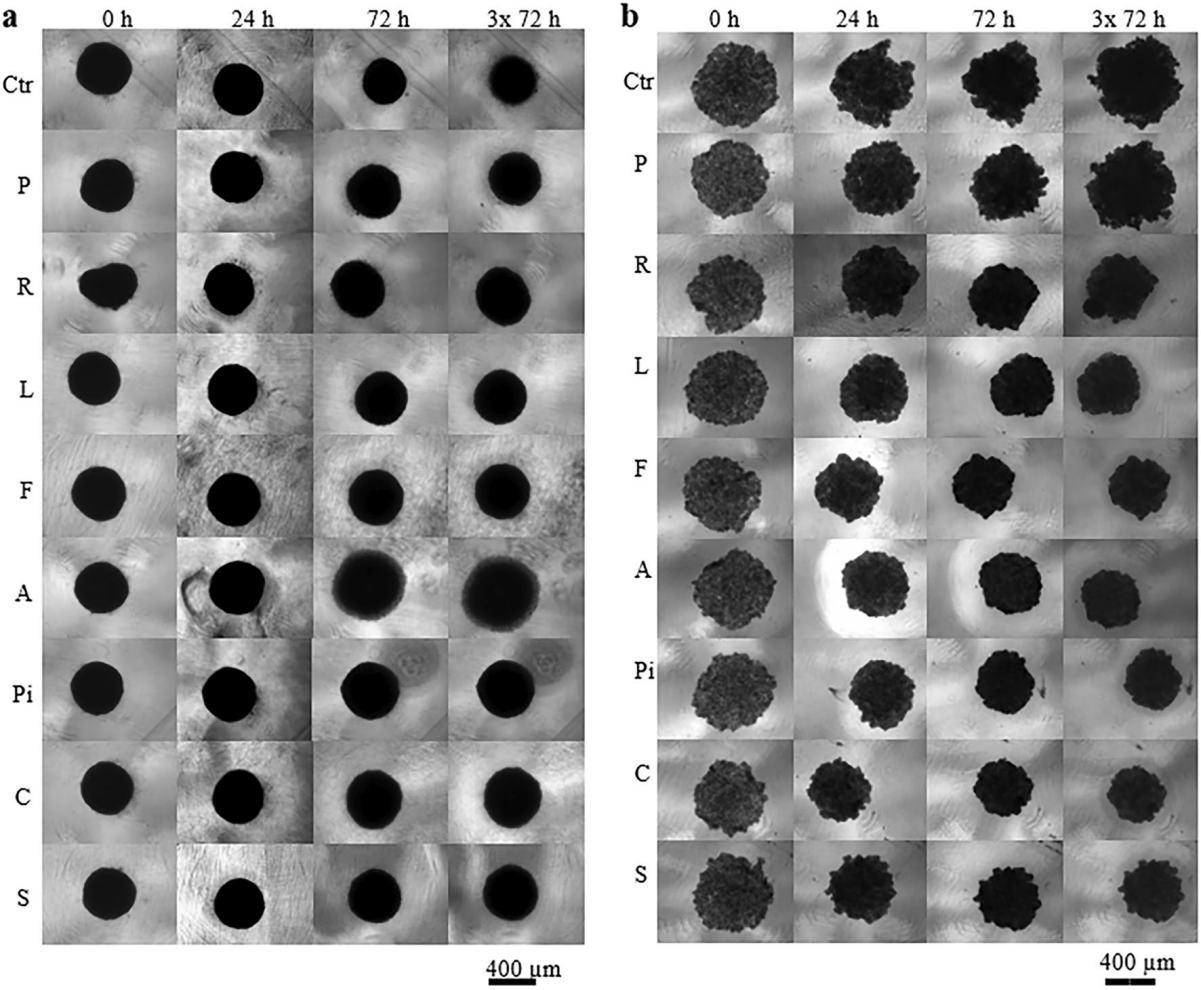
Effect of statins on the spheroid formation. (**a**) ADMSC stem cells, (**b**) pancreatic cancer MiaPaCa-2 cells, concentration of statins—20 µM, *Ctr* methanol treated spheroids, *P*—pravastatin, *R*—rosuvastatin, *L*—lovastatin, *F*—fluvastatin, *A*—atorvastatin, *Pi*—pitavastatin, *C*—cerivastatin, *S*—simvastatin. Statins were added once, 24 h after cell inoculation. Experiment was carried out in biological dodecaplicates.

The formation of spheroids of pancreatic cancer cells in short-time cultivation experiment (statins were added 24 h after cells inoculation) was affected by all the tested statins except for pravastatin (Fig. 4b). Only atorvastatin induced a change in stem cell spheroid formation that could be observed by light microscopy (Fig. 4a).

### Comparison of in vitro effects of statins on 2D and 3D experimental cancer and stem cells models

The effect of statins on the formation of spheroids from MiaPaCa-2 cells corelated with the effect of statins on the viability of cells growing in monolayers. Pravastatin (20 µM) had no effect on viability of MiaPaCa-2 cancer cells growing in a monolayer (Fig. 2) as well as on formation of spheroids (Fig. 4b). All other statins reduced the size of MiaPaCa-2 spheroids (Fig. 4b), that corelated with the inhibitory effect of statins on viability of MiaPaCa-2 cancer cells growing in a monolayer (Fig. 2).

We observed a delayed effect of statins (20 µM) on ADMSC stem cells grown in a monolayer (Fig. 2), only pravastatin was not effective. However, statins did not affect the formation of ADMSC spheroids; the only exception was atorvastatin, whose action resulted in the magnification of spheroids compared to the control ones (Fig. 4a).

The effect of statins on the size and compactness pre-formed MiaPaCa-2 cell spheroids was exactly opposite to their effect on the viability of cells grown in a monolayer. Interestingly, statins that were ineffective or only slightly effective in the 2D arrangement (pravastatin, rosuvastatin, atorvastatin, fluvastatin) (Fig. 2) had the most visible effect on the compactness of the spheroids (Fig. 3b). On the contrary, the effect of statins on the size and compactness of pre-formed spheroids of ADMSC stem cells (Fig. 3a) correlated well with the effect of statins on the viability of ADMSC stem cells grown in a monolayer (Fig. 2). In both experimental ADMSC models (2D and 3D), all the statins, except for rosuvastatin and pravastatin, were effective.

### Comparison of the in vitro effect of statins on the gene expression of pancreatic cancer MiaPaCa-2 cells and ADMSCs stem cells.

The transcriptional microarray analysis was used to study the effect of statins on the gene expression of ADMSC stem cells cultured for 24 h in 2D conditions in the presence of statins. Statins were administered at a concentration of 12 µM, which corresponds to the simvastatin IC_50_ value for MiaPaCa-2 cells after 24 h, used in the study of the effect of statins on gene expression of MiaPaCa-2 cells^11^.

Pravastatin-treated cells had the same transcription profile as control cells. Other statins significantly changed the transcription profile compared to that of control cells, of which rosuvastatin had the weakest effect (repository number *E-MTAB-11579)*.

**Figure 5:**
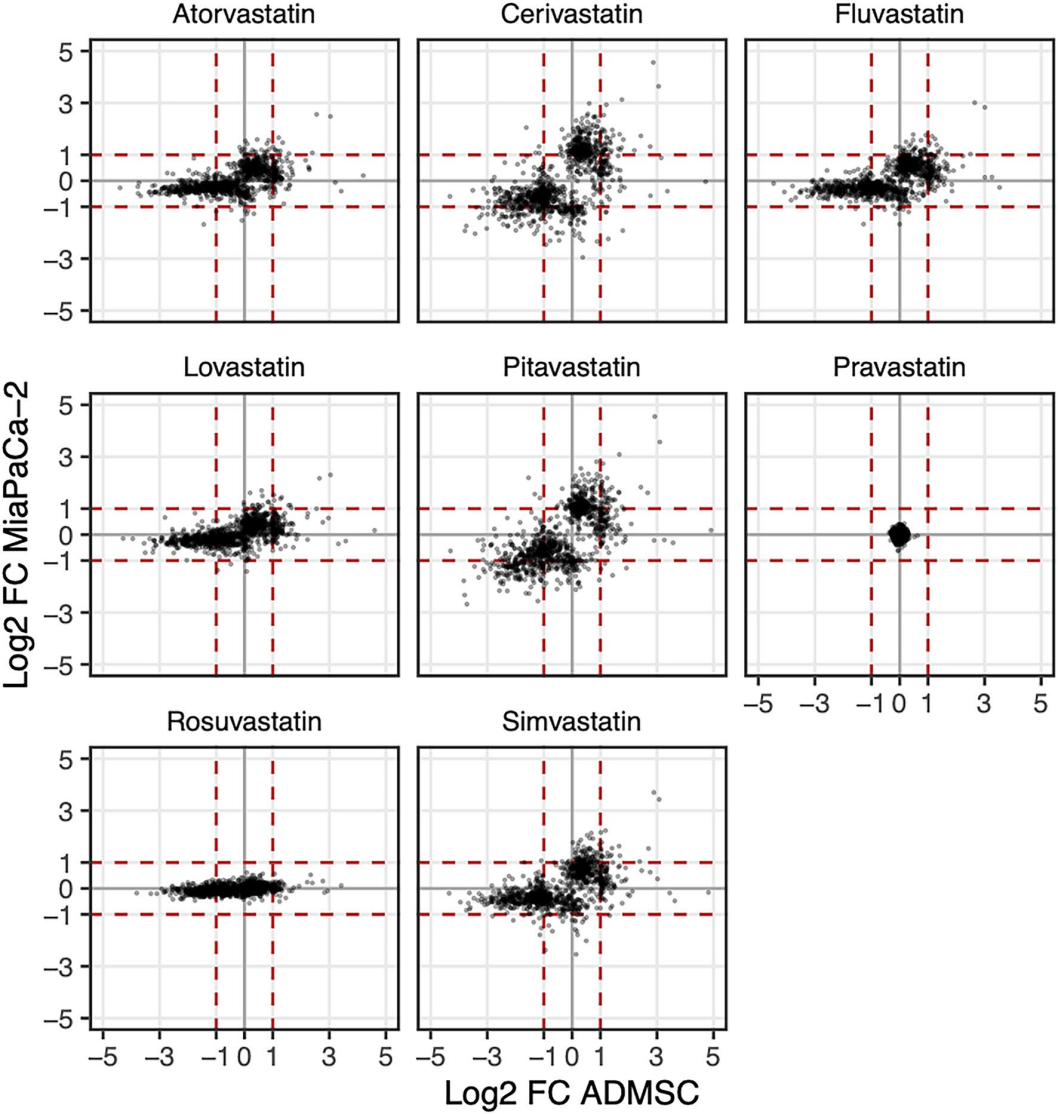
Comparison of expression changes between statin treated and control MiaPaCa-2 and ADMSC cells. Displayed are only the genes that are differentially expressed upon at least one statin treatment in at least one cell type, requiring |log_2_FC|> 1 and FDR < 0.05. Statins were administered at a concentration of 12 µM for 24 h. (*FC* fold change, *FDR* false discovery rate, horizontal and vertical axes—changes in ADMSC and MiaPaCa-2 cells, respectively, upon respective treatment). The red dashed lines indicate two-fold change increase or decrease in the gene expression. The genes with at least two-fold up-regulation (resp. down-regulation) in ADMSC stem cells are displayed to the right (resp. left) of the dashed lines. Similarly, genes with at least two-fold up-regulation (resp. down-regulation) in cancer cells are displayed above (resp. below) of the dashed lines. For details about differentially regulated transcripts see the ArrayExpress database, accessions *E-MTAB-3979, E-MTAB-11579*.

Comparison of the effect of statins on the gene expression of pancreatic cancer MiaPaCa-2 cells and ADMSCs stem cells is shown in Fig. 5.

In general, the trend of the effect of statins on the gene expression related to their lipophilicity is conserved in both cancer and stem cells^12^, specifically, the gene expression increased gradually with the lipophilicity of statins. The exception was the effect of rosuvastatin: rosuvastatin did not affect the gene expression of pancreatic cancer cells, while its effect on the transcription of stem cell genes was statistically significant (Fig. 5). Statins induced more frequently the gene down-regulation than up-regulation in stem cells, the trend was opposite in cancer cells (Fig. 5).

**Figure 6:**
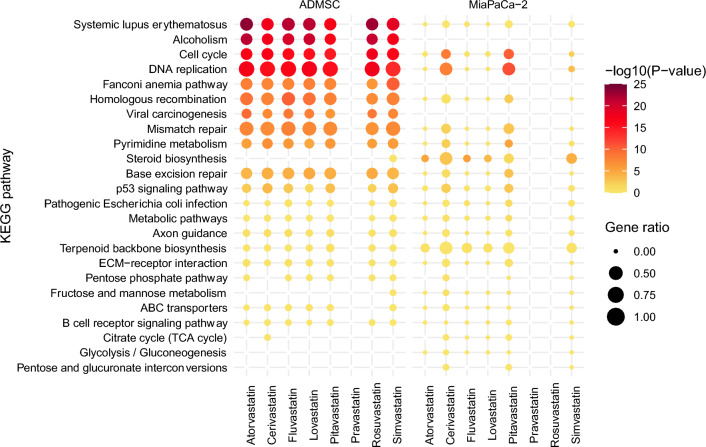
Cellular pathways most significantly affected by statins in cancer and stem cells. The gene set enrichment analysis (GSEA) revealed the KEGG pathways most affected by statin treatment in ADMSC and MiaPaCa-2 cells. Displayed is the union of the top five most enriched pathways among the comparisons. (Statin concentration—12 µM, treatment time—24 h, p-value—GSEA p-value, gene ratio—fraction of KEGG pathway genes among differentially expressed genes). For details about differentially regulated transcripts, see the ArrayExpress database, accessions *E-MTAB-3979, E-MTAB-11579.*

Comparison of the most significantly affected cellular pathways (according to KEGG) induced by statins in the cell lines studied is shown in Fig. 6.

**Figure 7:**
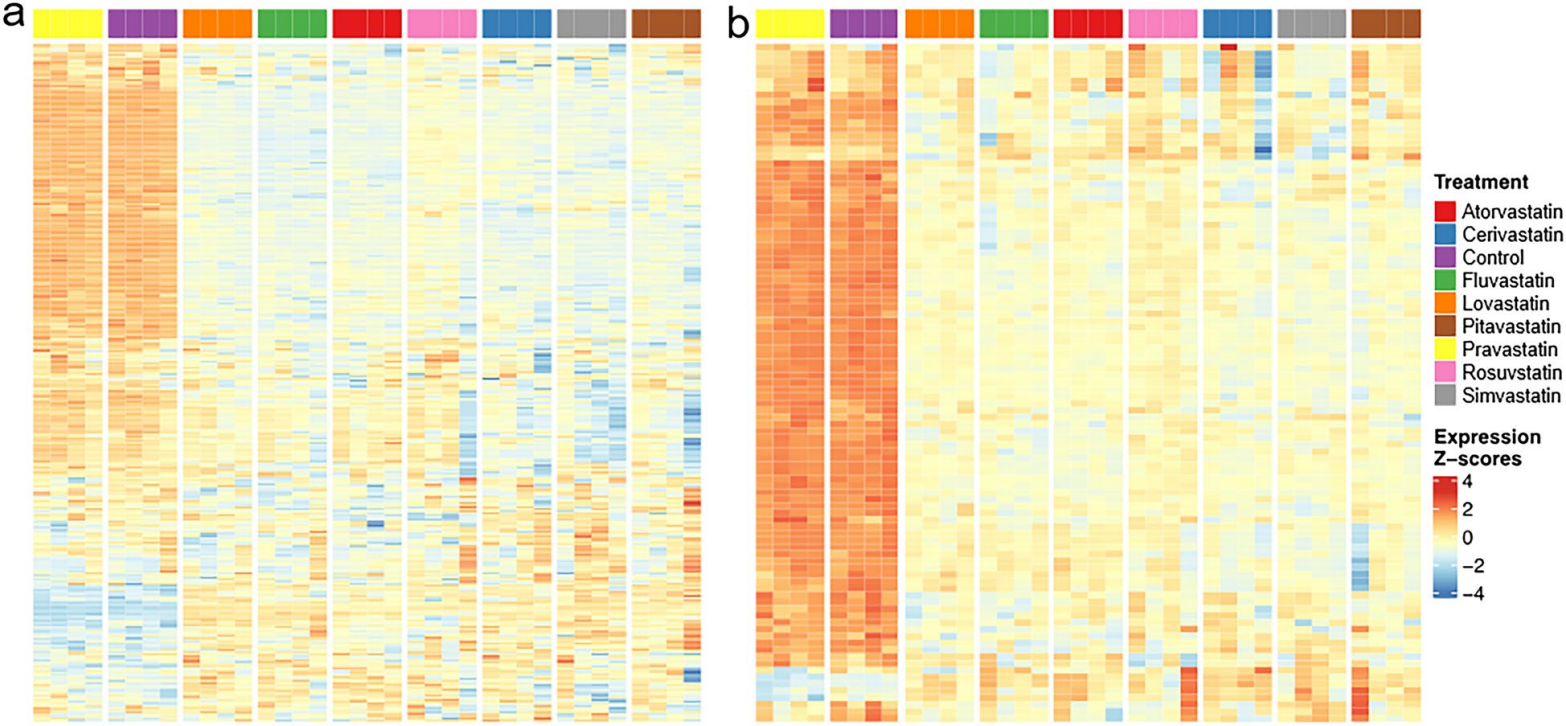
The effect of statins on (**a**) cell cycle, (**b**) DNA replication in ADMSCs. Figure represents heatmaps of z-score of the log expression intensities of differentially expressed genes. Presented are only the genes with statistically significant difference (FDR < 0.05) in expression intensity in at least one comparison statin vs. control and at least two-fold change of the expression intensity after the statin exposure. ADMSD—human adipose-derived mesenchymal stem cells, concentration of statins—12 µM, treatment time—24 h, the gene sets are based on KEGG pathways hsa04110 (cell cycle) and hsa03030 (DNA replication). For full list of differentially regulated transcripts see the ArrayExpress database, accession *E-MTAB-11579.*

In stem cells, statins significantly affected the cell cycle (Fig. 7a), especially decreased the expression of genes encoding several cyclin-dependent kinases (*E-MTAB-11579)*. Significant effect of statins on DNA replication (Fig. 7b) is related to the decrease of transcription of genes encoding the components of the PCNA complex, DNA polymerases α, δ, ε, helicase and DNA ligase, that indicates cell cycle arrest in S phase (*E-MTAB-11579)*.

## Discussion

Although the antiproliferative effect of statins on various cancers has been reported in a large number of studies, the effect of statins on stem cell viability has been published in only a few articles^13–18^. Furthermore, in most studies, the properties of all statins available on the market have not been collectively studied under identical conditions. The current paper and our previous reports^11,12,19,20^ are based on the evaluation of statins using the same experimental model under the same conditions. Statins in concentrations corresponding to those achieved with daily doses of statins in the treatment of cardiovascular diseases (lovastatin 20–80 mg, simvastatin 10–40 mg, fluvastatin 20–80 mg, pravastatin 10–80 mg, atorvastatin 10–80 mg, rosuvastatin 5–40 mg^21^) were used in our in vitro assays. Such concentrated solutions of statins (5–40 μM) with proven anti-proliferative effect on pancreatic cancer cells in vitro after 24 h of exposure^11^ did not affect the proliferation of the HEK 293 non-cancerous cells and ADMSC stem cells. We were unable to observe any effect of statins on stem cells even at a concentration of 100 μM after a time period of 24 h. Similar concentrations (30–60 μM) were used in study by Izadpanah et al., where they observed a significant increase in the doubling time of mesenchymal stem cells exposed to pravastatin and atorvastatin during the initial two passages^22^.

Some statins (e.g. lovastatin) belong to substances whose IC_50_ values decrease significantly by lowering the pH from 7.5–7.7 to 6.7–6.8^23,24^. Therefore, in chemoadjuvant therapy of specific types of tumors characterized by local pH reduction, statins could be used even at lower doses than in hypercholesterolemia treatment. Therefore, the antiproliferative or pleiotropic effects of statins on non-cancerous and stem cells would be minimal.

Studies investigating changes in gene expression induced by statin treatment using microarray technology have been published since 2000^25,26^. These results are difficult to compare due to the usage of different statins, different statin concentrations, or experimental models. Moreover, in the case of the array analysis, the results are difficult to compare even between our experiments. Although we used the same tested concentration of all statins for both ADMSCs and pancreatic cancer cells (12 μM, 24 h), the microarray platform was changed between experiments. As the probes design and sensitivity of platforms differ, the absolute number of genes whose expression was affected, as well as the intensity of changes in the expression of individual genes is not entirely comparable. However, it is obvious that the trend in the effectivity of statins on the gene expression related to their lipophilicity is preserved both in tumor and stem cells^12^. The only exception is rosuvastatin, which was ineffective at the concentration tested in pancreatic cancer cells; however, its effect on stem cell gene transcription was statistically significant (Fig. 5).

Atorvastatin, simvastatin, fluvastatin, lovastatin, cerivastatin, and pitavastatin are relatively lipophilic, while rosuvastatin and pravastatin are hydrophilic. While hydrophilic statins cannot easily pass through the cell membranes and are taken up mainly by hepatocytes, which are also the target of statin treatment from a therapeutic point of view, lipophilic statins, due to their easier passage through cell membranes, also reach other types of cells^27^. The question is whether rosuvastatin enters stem cells more easily compared to tumor cells in general or only to adipose tissue derived stem cells. Based on our data and many other published reports, it seems that the biological effects of statins are cell specific, dependent on the individual statins used, and dependent on the concentration reached within the specific cell compartment.

The cell cycle arrest of ADMSCs stem cells is indicated not only by data obtained by the microarray analysis, but also by light microscopy images. Under the microscope, we saw a greater number of round cells in the statin-affected cell population than in the statin-unaffected control cell population (data not shown). Moreover, after the removal of statins from the medium, during long-term cultivation, the cells began to divide again. Lovastatin has been reported to inhibit the transition from G1 to S phase of the cell cycle^28,29^ and is the only statin that synchronizes cell populations in the G1 phase by inhibiting the proteasome^30^. However, we observed differences in the influence of individual statins and also in the way in which statins affected the cell cycle of different types of pancreatic cancer cells. In the case of MiaPaCa-2 cells (human pancreatic tumor cells with an activating mutation in the *KRAS* gene), the two most effective statins, pitavastatin and cerivastatin, had the most significant effect on the cell cycle; an increase in the number of cells was evident in the G0/G1 phase. Lovastatin also had a comparable effect. As the rate of cell differentiation increased, the importance of the effect of statins on the cell cycle of cells decreased. In more differentiated cell lines (BxPC-3, CAPAN-2), there was an increase in the number of cells in the G2/M phase^31^. In the case of ADMSCs, we observed that the expression of genes related not only to the cell cycle but also to DNA replication and DNA repair mechanisms was statistically significantly changed (Fig. 6).

This could support our hypothesis that even in stem cells, statins inhibit the cell cycle in the G1 phase. The significant influence of statins on the expression of genes related to DNA repair mechanisms calls for refuting their possible genotoxic effects, caused either through the induction of reactive oxygen intermediates, or by inhibition the farnesylation of proteins that appear directly during the division and re-synthesize of the nucleus, to which belong, for example, lamins^32^.

The presented results open up many other questions that should be answered before alternative use of statins in medicine. Although we observed changes in spheroid size, compactness, and formation, we do not know the causes or mechanisms by which statins induced these changes, as there are currently no cost-effective methodological protocols for quantifying spheroid changes providing reproducible results for screening studies. A comparison of the influence of individual statins on non-cancer and cancer stem cells, that represent the most resistant population and eventually give rise to tumor growth would be very interesting, however, due to the scope of the experiments, it is for a separate study. We interpreted only a part of the results of the whole transcriptome microarray assay, most of them await the interpretation and subsequent confirmation of the hypotheses based on them with further experiments.

## Conclusions

Non-cancerous and stem cells tested are more resistant to the effects of statins compared to cancer cells, especially after the first 24 h of treatment. The trend in intensity of the effect of individual statins on the gene expression of ADMSD stem cells correlated with published data on MiaPaCa-2 cells. The exception was rosuvastatin, which affected the gene expression in ADMSC stem cells, while it had no effect on gene expression in pancreatic cancer MiaPaCa-2 cells. Due to the low efficacy of statins on non-tumor and stem cells at concentrations suitable for tumor treatment, our data support the suitability of statins in the chemoadjuvant tumor therapy. However, substantially distinct are not only the effects of statins on cancerous, non-cancerous and stem cells in vitro, but also on individual types of cells growing in 2D and 3D arrangement. For the use of statins as chemoadjuvants, it will be also necessary to investigate the consequences of the induction of spheroid disintegration by statins to rule out possible promotion of tumor metastasis.

## Materials and methods

### Materials

In all experiments, pure forms (≥ 98%) of the following statins were used: atorvastatin (A7658), lovastatin (M1687), simvastatin (S3449), fluvastatin (F4482), cerivastatin (C1668), pravastatin (P6801), rosuvastatin (R5974), and pitavastatin (P3576) (LKT Laboratories, USA). All statins were dissolved in pure methanol (Centralchem, s.r.o., Slovakia) to concentration of 20 mM and stored at − 20 °C. The final concentration of methanol never exceeded 1%.

### Cell cultures

The human pancreatic cancer cell line MiaPaCa-2 (ATCC, Manassas, VA, CRL-1420, LOT 70014313, RRID: CVCL_0428) and human embryonic kidney cell line HEK 293 (ATCC, Manassas, VA, CRL-1573, LOT 70039815, RRID:CVCL_0045) were cultured in Dulbecco's Modified Eagle Medium (DMEM; Sigma Aldrich, Germany) supplemented with 10% fetal bovine serum. Human adipose-derived mesenchymal stem cells ADMSC (ATCC, Manassas, VA, PSC-500-011, LOT 70017032, positive specific staining for CD29, CD44, CD73, CD90, CD105, and CD166 and negative for CD14, CD31, CD34, and CD45) were cultured in mesenchymal stem cell basal medium (ATCC, Manassas, VA) supplemented with low serum mesenchymal stem cell growth kit for adipose- and umbilical-derived MSCs (ATCC, Manassas, VA). All cell lines were kept in a humidified atmosphere containing 5% CO_2_ at 37 °C.

### Evaluation of cell growth and viability under 2D conditions

The effects of individual statins (pravastatin, atorvastatin, simvastatin, lovastatin, cerivastatin, rosuvastatin, and fluvastatin) on the viability of human pancreatic cancer MiaPaCa-2 cells reported in our previous studies^11,12^ were compared with their effects on non-cancerous HEK 293 and ADMSC stem cells. The amounts of 10^4^ and 3.5 × 10^3^ of HEK 293 cells and ADMSC (passage 8), respectively, were used for inoculation of individual wells in 96-well plates (TPP, Switzerland). After 24 h of incubation, the cells were treated with individual statins at 10, 20, 30, 40, 50, 60, 80, and 100 µM concentration dissolved in fresh cell culture media. Untreated cells and cells treated only with solvent (1% methanol) at the corresponding amount served as controls. After 24, 48, and 72 h, the WST-1 cell viability test was performed spectrophotometrically (BioTek ELx808™ Plate reader, Lonza) and evaluated with Gen5 software. Data are presented as mean ± SD.

### Cell growth and viability assessment in 3D condition

The U-shaped surface of 96-wells (Guangzhou Jet Bio-Filtration Co., Ltd., China) was covered with a microlayer of SeaKem LE® agarose (Lonza, Switzerland). Two different approaches were applied to investigate the effect of statins on the growth and compactness of cell spheroids.

#### Long-term cultivation

The first approach evaluated the effect of statins on the size, shape, and compactness of the spheroids. For this purpose, 2 × 10^3^ and 10^3^ ADMSC and MiaPaCa-2 cells, respectively, were used for inoculation of individual wells of the agarose adjusted U-shaped 96-well plates. During the spheroids’ formation, 30 µL of cell culture medium was added to each well every 8th day. The ADMSC and MiaPaCa-2 cells formed the spheroids within 10 and 3.5 weeks, respectively. Subsequently, each spheroid was transmitted individually into new well and treated with individual statins at 20 µM concentration. The size, shape, and spheroids´ compactness were evaluated by light microscope (Zeiss Axio Vert.A1, Carl Zeiss Microscopy GmbH, Germany) with photo documentation equipment (CCD camera Axiocam ICC 1 with Axio Vision 4.8 software, Carl Zeiss Microscopy GmbH, Germany) before statins addition and subsequently after the addition of statins three times at 72-h intervals.

#### Short-term cultivation

The second approach evaluated the effect of individual statins on the formation of spheroids. The amount of 8.5 × 10^4^ and 4.5 × 10^4^ of ADMSC and MiaPaCa-2 cells, respectively, was used for inoculation of individual wells of the agarose adjusted U-shaped 96-well plates. Statins at 20 µM concentration were added 24 h after cell inoculation. The effect of statins on spheroid formation was observed by a light microscope with a photo documentation device after 24, 48, and 72 h and two more times at 72 h intervals.

The experiments were carried out in biological dodecaplicates. The method enables only to compare the morphology of the treated spheroids with the control ones, not to assess the reasons for the observed changes in morphology or to quantify the microscopically visible/detectable changes.

### Transcriptional microarray analysis

For stem cell transcriptional microarray analysis, ADMSCs (3.5 × 10^5^ cells per dish, passage 8) were used for the inoculation of a 6-cm^2^ cell culture dish (TPP, Switzerland) in triplicate (total media volume of 6 mL). After 24 h of incubation, the cells were treated with a 12 µM concentration of statins (corresponding to simvastatin IC_50_ value for MiaPaCa-2 cells after 24 h). Cells treated with fresh cell culture medium (untreated cells) or cells treated with only the solvent at the corresponding amount (methanol) served as controls. The cells were further incubated for 24 h. Then, the cells were lysed in the stage of subconfluency using 0.15 mL of the RLT lysis buffer supplied in RNeasy Micro Kit (Qiagen, USA). Total ribonucleic acid (RNA) was isolated using RNeasy Micro Kit (QIAGEN, USA) from 0.35 mL of cell lysate. The quantity and quality of RNA were ascertained using NanoDrop ND-1000 spectrophotometer (NanoDrop Technologies LLC, USA) and Agilent 2100 Bioanalyzer (Agilent Technologies, CA, USA). All samples that had the RNA integrity number (RIN) greater than 7 were used for microarray analysis of the Clairom S Assay (Applied Biosystems, Waltham, MA, USA) following the standard protocol: A total of 250 ng of RNA were amplified and fragmented using the kit provided. Then, 2300 ng of the resulting fragmented cDNA was hybridized on the chips according to the manufacturer’s protocol. Each sample group was evaluated in four biological replicates.

The resulting CEL files were processed using the packages oligo^33^ and limma^34^ of the Bioconductor^35^ within the R environment^36^: robust multiarray analysis^37^ was used to standardize the data and moderated t-test was used to detect differentially expressed transcripts after fitting linear model I ~ Treatment. Storey’s q-value less than 0.05^38^ and a minimally two-fold change in expression intensity were required to consider differentially transcribed genes. The minimum information about a microarray experiment (MIAME) compliant data was deposited to the ArrayExpress database (repository number *E-MTAB-3979, E-MTAB-11579*).

The hypergeometric test was used to analyze the over-representation of gene sets defined by KEGG (Kyoto Encyclopedia of Genes and Genomes) pathways^39^ and the terms of the GO (Gene Ontology)^40^ represented by at least 5 expressed genes. For GO gene sets, we reported only terms exhibiting an over-representation p-value lower than 0.001.

The workflow and results of whole-genome microarray analysis of MiaPaCa-2 cells treated with individual statins has been published in our previous study^12^. The microarray platform was changed between experiments since a longer period has passed between them and new possibilities of analysis have appeared. Illumina HumanWG-6_V3 Expression BeadChips (Illumina, CA, USA) and Clairom S Assay (Applied Biosystems, Waltham, Massachusetts, USA) were used to perform the experiment on MiaPaCa-2 and ADMSC cells, respectively.

### Quantitative real-time PCR

Validation of the selected gene expression changes was performed using quantitative RT-PCR. The cells cultivation, their treatment and lysis, RNA isolation and its quantification for qPCR and microarrays analyses were performed as above. Reverse transcription was performed with the QuantiTect reverse transcription kit (QIAGEN Inc., Germantown, MD, USA). qPCR was performed on the LightCycler 2.0 System using LightCycler 480 DNA SYBR Green I Master kit (Roche Diagnostics, Basil, Switzerland) and the results were analysed by LightCycler software. Crossing point *C*_*P*_ values were further analysed using *ΔΔC*_*P*_ method^41^ within the R environment^36^. The *GAPDH*, *RPS9* and *TBP* genes (resp. *RPL13*, *HPRT1*, *PPIA*, and *TBP*) were used as reference genes in the analysis of MiaPaCa-2 cells (resp. ADMSC). A detailed description of the analysis and the list of amplicons/primers of target and housekeeping genes are provided in Supplementary Tables 1–4.

### Supplementary Information

Supplementary Tables.

## Data Availability

The datasets generated during and/or analysed during the current study are available in the ArrayExpress database (repository number *E-MTAB-3979* (https://www.ebi.ac.uk/biostudies/arrayexpress/studies/E-MTAB-3979), *E-MTAB-11579* (https://www.ebi.ac.uk/biostudies/arrayexpress/studies/E-MTAB-11579)). A detailed description of the experimental conditions of Quantitative real-time PCR and the list of amplicons/primers of target and housekeeping genes are provided in Supplementary Tables 1–4.

## References

[1] Lapteva L, Vatsan R, Purohit-Sheth T (2018). Regenerative medicine therapies for rare diseases. Transl. Sci. Rare Dis..

[2] Rajabzadeh N, Fathi E, Farahzadi R (2019). Stem cell-based regenerative medicine. Stem Cell Investig..

[3] Zakrzewski W, Dobrzyński M, Szymonowicz M, Rybak Z (2019). Stem cells: Past, present, and future. Stem Cell Res. Ther..

[4] Hwang NS, Varghese S, Elisseeff J (2008). Controlled differentiation of stem cells. Adv. Drug Deliv. Rev..

[5] Werbowetski-Ogilvie TE (2009). Characterization of human embryonic stem cells with features of neoplastic progression. Nat. Biotechnol..

[6] Park A (2016). Use of statins to augment progenitor cell function in preclinical and clinical studies of regenerative therapy: A systematic review. Stem Cell Rev. Rep..

[7] Goldstein JL, Brown MS (2015). A century of cholesterol and coronaries: From plaques to genes to statins. Cell.

[8] Alexandrova R (2019). Briefly about anticancer properties of statins. Biomed. J. Sci. Tech. Res..

[9] Mohammadkhani N (2019). Statins: Complex outcomes but increasingly helpful treatment options for patients. Eur. J. Pharmacol..

[10] Pisanti S, Picardi P, Ciaglia E, D’Allesandro A, Bifulco M (2014). Novel prospects of statins as therapeutic agents in cancer. Pharmacol. Res..

[11] Gbelcová H (2008). Differences in antitumor effects of various statins on human pancreatic cancer. Int. J. Cancer.

[12] Gbelcová H (2017). Variability in statin-induced changes in gene expression profiles of pancreatic cancer. Sci. Rep..

[13] Gorabi AM (2021). Effects of statins on the biological features of mesenchymal stem cells and therapeutic implications. Heart Fail. Rev..

[14] Lee H, Lee H, Na CB, Park JB (2019). The effects of simvastatin on cellular viability, stemness and osteogenic differentiation using 3-dimensional cultures of stem cells and osteoblast-like cells. Adv. Clin. Exp. Med..

[15] Pini R (2020). Different drugs effect on mesenchymal stem cells isolated from abdominal aortic aneurysm. Ann. Vasc. Surg..

[16] Li Y (2015). Statins impair survival of primary human mesenchymal progenitor cells via mevalonate depletion, NF-κB signaling, and Bnip3. J. Cardiovasc. Transl. Res..

[17] Xue D, Gong Z, Zhu F, Qiu Y, Li X (2018). Simvastatin increases cell viability and suppresses the expression of cytokines and vascular endothelial growth factor in inflamed human dental pulp stem cells in vitro. Adv. Clin. Exp. Med..

[18] Xu R, Chen J, Cong X, Hu S, Chen X (2008). Lovastatin protects mesenchymal stem cells against hypoxia-and serum deprivation-induced apoptosis by activation of PI3K/Akt and ERK1/2. J. Cell. Biochem..

[19] Gbelcová H (2013). The effect of simvastatin on lipid droplets accumulation in human embryonic kidney cells and pancreatic cancer cells. Lipids Health Dis..

[20] Rimpelová S (2021). Comparison of transcriptomic profiles of MiaPaCa-2 pancreatic cancer cells treated with different statins. Molecules.

[21] Grundy SM (2019). 2018 AHA/ACC/AACVPR/AAPA/ABC/ACPM/ADA/AGS/APhA/ASPC/NLA/PCNA guideline on the management of blood cholesterol: Executive summary: A report of the American College of Cardiology/American Heart Association Task force on clinical practice guidelines. Circulation.

[22] Izadpanah R (2015). The impact of statins on biological characteristics of stem cells provides a novel explanation for their pleiotropic beneficial and adverse clinical effects. Am. J. Physiol. Cell Physiol..

[23] Kobayashi M (2007). Effect of medium pH on the cytotoxicity of hydrophilic statins. J. Pharm. Pharm. Sci..

[24] Kobayashi H (2017). Cancer chemotherapy specific to acidic nests. Cancer.

[25] Jacobson JR (2004). Cytoskeletal activation and altered gene expression in endothelial barrier regulation by simvastatin. Am. J. Respir. Cell Mol. Biol..

[26] Johnson-Anuna LN (2005). Chronic administration of statins alters multiple gene expression patterns in mouse cerebral cortex. J. Pharmacol. Exp. Ther..

[27] Schachter M (2005). Chemical, pharmacokinetic and pharmacodynamic properties of statins: An update. Fundam. Clin. Pharmacol..

[28] Javanmoghadam-Kamrani S, Keyomarsi K (2008). Synchronization of the cell cycle using lovastatin. Cell Cycle.

[29] Ma HT, Poon RY (2017). Synchronization of HeLa cells. Methods Mol. Biol..

[30] Rao S (1999). Lovastatin- mediated G1 arrest is through inhibition of the proteasome, independent of hydroxymethyl glutaruyl-CoA reductase. Proc. Natl. Acad. Sci. USA.

[31] Režňáková, S. et al. Vplyv statínov na aktínový cytoskelet a bunkový cyklus pankreatických nádorových buniek. In *Biologicko-Genetické Aspekty Nádorovej Regeneračnej Medicíny* 12–16, (Martin JLF UK, 2016), ISBN 978-80-8187-021-7.

[32] Worman HJ, Courvalin JC (2000). The inner nuclear membrane. J. Membr. Biol..

[33] Durinck S (2005). BioMart and Bioconductor: A powerful link between biological databases and microarray data analysis. Bioinformatics.

[34] Hubbard TJ (2007). Ensembl 2007. Nucleic Acids Res..

[35] Carvalho BS, Irizarry RA (2010). A framework for oligonucleotide microarray preprocessing. Bioinformatics.

[36] R Core Team R. *A Language and Environment for Statistical Computing* (R foundation for statistical computing; 2023). https://www.R-project.org/.

[37] Ritchie ME (2015). limma powers differential expression analyses for RNA-sequencing and microarray studies. Nucleic Acids Res..

[38] Storey JD, Tibshirani R (2003). Statistical significance for genomewide studies. Proc. Natl. Acad. Sci. USA.

[39] Kanehisa M, Furumichi M, Tanabe M, Sato Y, Morishima K (2017). KEGG: New perspectives on genomes, pathways, diseases and drugs. Nucleic Acids Res..

[40] Gene Ontology Consortium (2015). Gene Ontology Consortium: Going forward. Nucleic Acids Res..

[41] Pfaffl MW (2001). A new mathematical model for relative quantification in real-time RT-PCR. Nucleic Acids Res..

