# Supplementary material for: Highly variable biological effects of statins on cancer, non-cancer, and stem cells in vitro
Source: Sci Rep. 2024 May 23;14:11830. doi: 10.1038/s41598-024-62615-w (PMC11116523; doi:10.1038/s41598-024-62615-w)
Supplement: Supplementary file 1 — Supplementary Tables. [file 41598_2024_62615_MOESM1_ESM.docx]

**HIGHLY VARIABLE BIOLOGICAL EFFECTS OF STATINS ON CANCER AND NON-CANCER CELLS *IN VITRO***

**Cellular and Molecular Life Sciences**

***Helena Gbelcová, Silvie Rimpelová, Adriana Jariabková, Patrik Macášek, Petra Priščáková, Tomáš Ruml, Jana Šáchová*, *Jan Kubovčiak, Michal Kolář, Libor Vítek***

***corresponding authors:***

Helena Gbelcová, Institute of Medical Biology, Genetics and Clinical Genetics, Faculty of Medicine, Comenius University, Bratislava, Slovak Republic, [helena.gbelcova@fmed.uniba.sk](mailto:helena.gbelcova@fmed.uniba.sk)

**Supplementary Table 1.** List of primers used for quantitative real-time PCR analyses (MiaPaCa-2 cells)

**Supplementary Table 2.** Quantitative PCR analysis of selected genes (MiaPaCa-2 cells)

Fold expression changes in statin treated samples vs. controls as detected in RT-qPCR and microarray analyses. Figures in bold denote statistically significant changes (p < 0.1 for RT-qPCR, and FDR < 0.1 for microarray data). The symbols denote: n.a. not available, n.s. not significant. p (resp. FDR) < 0.1, * p < 0.05, ** p < 0.01, *** p < 0.001.

**Supplementary Table 3.** List of primers used for quantitative real-time PCR analyses (ADMSC cells)

**Supplementary Table 4.** Quantitative PCR analysis of selected genes (ADMSC cells)

Fold expression changes in statin treated samples vs. controls as detected in RT-qPCR and microarray analyses. Figures in bold denote statistically significant changes (p < 0.1 for RT-qPCR, and FDR < 0.1 for microarray data). The symbols denote: n.a. not available, n.s. not significant. p (resp. FDR) < 0.1, * p < 0.05, ** p < 0.01, *** p < 0.001.
